# Critical Care Monitoring in Comatose Patients Receiving ECMO: A Cohort Study of Machine Learning on Quantitative EEG for Diagnosing Acute Brain Injury and Prognosticating Mortality

**DOI:** 10.3390/jcm15176761

**Published:** 2026-08-31

**Authors:** Mingfeng Cao, Jeffrey B. Wang, Beichen Shen, Zoe Soule, Kotaro Noda, Jaeho Hwang, Eva Ritzl, Yaman B. Ahmed, Hyun-Yi Woo, Siyu Wang, Tianyue Zhu, Leon Fan, Nirma Carballido Martinez, Glenn Whitman, Nitish Thakor, Sung-Min Cho

**Affiliations:** 1 Department of Biomedical Engineering, Whiting School of Engineering, Johns Hopkins University, Baltimore, MD 21218, USA; mcao10@jhu.edu (M.C.); bshen9@jh.edu (B.S.); thakorjhu@gmail.com (N.T.); 2 Division of Neuroscience Critical Care, Departments of Neurosurgery, Anesthesiology and Critical Care Medicine, The Johns Hopkins Hospital, Baltimore, MD 21287, USA; jwang600@jh.edu (J.B.W.); zsoule1@jhu.edu (Z.S.); knoda2@jh.edu (K.N.); yahmad5@jh.edu (Y.B.A.); hwoo12@jh.edu (H.-Y.W.); swang333@jh.edu (S.W.); 3 Department of Neurology, Hospital for Special Surgery, New York, NY 10021, USA; 4 Department of Neurology, Northwestern University Feinberg School of Medicine, Chicago, IL 60611, USA; jhwang@nm.org; 5 Department of Neurology, Massachusetts General Hospital, Boston, MA 02114, USA; eritzl@mgh.harvard.edu; 6 Department of Neurology, Johns Hopkins University School of Medicine, Baltimore, MD 21205, USA; ncarbal1@jhmi.edu; 7 Division of Cardiac Surgery, Department of Surgery, Johns Hopkins University School of Medicine, Baltimore, MD 21287, USA; tzhu17@jh.edu (T.Z.); lfan17@jh.edu (L.F.); gwhitman@jhmi.edu (G.W.)

**Keywords:** quantitative EEG, extracorporeal membrane oxygenation, acute brain injury, machine learning, SHAP, neuromonitoring, neuroprognostication

## Abstract

**Background:** Acute brain injury (ABI) is a major cause of mortality and morbidity during extracorporeal membrane oxygenation (ECMO), yet early diagnosis remains challenging because neuroimaging is often impractical in critically ill patients. We evaluated whether quantitative electroencephalography (qEEG) combined with machine learning could identify ABI and predict mortality in patients receiving ECMO. **Methods:** Consecutive adult ECMO patients who underwent a standardized neuromonitoring protocol with continuous EEG during sedation interruption were retrospectively analyzed. Quantitative EEG features and clinical variables were extracted and used to train multiple machine-learning classifiers with leave-one-subject-out cross-validation. **Results:** Fifty-seven patients were included (mean age 56 years; 54% male), including 41 supported with venoarterial ECMO, 15 with venovenous ECMO, and one with venoarterial-venous ECMO. ABI occurred in 21 patients (37%), of whom 70% had ischemic injury. Models incorporating qEEG achieved higher point estimates than those using clinical variables alone for ABI detection (best area under the curve (AUC) 0.769, 95% confidence interval (CI) 0.638–0.883, vs. 0.681), although the difference did not reach statistical significance. Frontal theta power and interhemispheric asymmetry were the EEG features most strongly associated with ABI. qEEG features also carried prognostic information for 30-day mortality (best AUC 0.864). **Conclusions:** Machine-learning analysis of continuous qEEG acquired during standardized sedation interruption may provide a noninvasive bedside approach for identifying ECMO patients at increased risk of ABI and short-term mortality and may help prioritize urgent neuroimaging and neurological intervention.

## 1. Introduction

Extracorporeal membrane oxygenation (ECMO) is a rescue therapy for patients with refractory cardiac or respiratory failure. Its clinical use has expanded substantially in recent years, with demonstrated survival benefits across multiple indications [1,2,3,4]. However, neurological complications remain a major source of morbidity and mortality during ECMO support [5,6,7,8]. Acute brain injury (ABI) represents a major complication in patients receiving ECMO [9,10], while detection remains challenging as neurological examinations are frequently limited by sedation, paralysis, and critical illness. Systemic metabolic and hemodynamic disturbances may further confound accurate assessment of underlying brain injury [10].

Currently, ABI detection in ECMO relies largely on neuroimaging, which requires transport of hemodynamically unstable patients receiving mechanical circulatory support, exposing patients to risks such as hemodynamic deterioration, interruption of life-sustaining therapies, circuit complications, and accidental decannulation [11,12,13]. Consequently, a substantial proportion of ECMO patients never undergo head computed tomography (CT) during their support. Conventional magnetic resonance imaging (MRI) is frequently impractical because of ECMO hardware, monitoring equipment, patient instability, and logistical barriers to transport [14,15,16]. Although new technologies such as ultra-low-field portable MRI have emerged as a promising bedside alternative for ECMO patients, their availability remains limited and adoption has not yet become widespread [17,18]. Moreover, imaging only provides a static snapshot, limiting the ability to monitor evolving injury. There is a critical unmet need for continuous bedside monitoring approaches capable of detecting ABI in patients supported with both venoarterial (VA) and venovenous (VV) ECMO.

Electroencephalography (EEG) offers a compelling alternative by providing continuous, real-time assessment of cerebral electrophysiology at the bedside without patient transport [19], and can reveal dynamic, potentially early signatures of ABI before structural changes become apparent. Continuous EEG is increasingly recommended in ECMO patients [12,16,20], but its interpretation relies on expert visual review, whereas quantitative EEG (qEEG) yields objective, reproducible metrics of cerebral function [21,22,23]. Because these metrics are high-dimensional, spanning many channels, frequency bands, and time points, they are well suited to machine learning. In ECMO, machine-learning models have predicted ABI from clinical and hemodynamic data [24], but these are systemic surrogates and do not capture direct measures of brain function. Applying machine learning to qEEG could recover this brain-specific signal and provide an automated, reproducible bedside approach for detecting evolving ABI.

In this study, we hypothesized that machine learning applied to qEEG obtained during sedation interruption would improve the detection of clinically significant ABI and predict 30-day mortality in comatose patients supported with ECMO. To test this hypothesis, we retrospectively studied comatose adults supported on VA- and VV-ECMO who underwent continuous EEG during protocolized sedation interruption. We extracted a broad panel of quantitative EEG features, trained and evaluated multiple machine-learning classifiers under leave-one-subject-out cross-validation (LOSO-CV), and used interpretability analysis to identify the EEG features most associated with each outcome metric.

## 2. Materials and Methods

### 2.1. Study Design

This was a single-center, retrospective cohort study at the Johns Hopkins Hospital, an academic tertiary referral center, approved by the Johns Hopkins Medicine Institutional Review Board with a waiver of informed consent given the retrospective design and use of de-identified data (IRB00216321). The study was conducted and reported in accordance with the STROBE statement for observational studies and the TRIPOD+AI statement for prediction model development [25,26].

### 2.2. Study Population

Adult patients (age ≥ 18 years) supported on ECMO at the Johns Hopkins Hospital between November 2017 and September 2024 were retrospectively identified from the institutional ECMO registry and EEG monitoring service records. At our institution, all ECMO patients are followed by a dedicated neurocritical care consult team from the day of cannulation through hospital discharge or death [12,13]. A baseline neurological examination is documented, and daily sedation holidays are attempted from day 3 to 5, with continued weaning thereafter.

Continuous EEG (cEEG) monitoring is initiated in patients meeting any of the following criteria: a Glasgow Coma Scale (GCS) ≤ 8 off sedation with clinical stability for at least 24 h; cardiac arrest as the ECMO indication, with cEEG initiated within 72 h of cannulation; or any clinical concern for seizures. Patients with GCS > 8 off sedation or intraoperative-only ECMO support were not monitored. cEEG monitoring is continued for at least 24 h when clinically tolerated. Eligible patients were comatose adults on ECMO with at least one continuous EEG recording of ≥12 uninterrupted hours and qualifying brain neuroimaging. Full inclusion and exclusion criteria are provided in the Supplementary Materials.

### 2.3. Clinical and Demographic Data Collection

Clinical, demographic, and laboratory data were extracted from the institutional electronic health record using a standardized case report form. Demographic and ECMO-related variables included age, sex, race, ECMO configuration (VA, VV, or VA-V), cannulation site, primary ECMO indication (cardiac or pulmonary support), pre-ECMO cardiac arrest including extracorporeal cardiopulmonary resuscitation (ECPR), sedation regimen at EEG initiation, GCS score, and Richmond Agitation–Sedation Scale (RASS) score at EEG initiation.

Laboratory data were extracted at two time points: T1 (closest to EEG initiation) and T2 (24 h later), comprising the complete blood count, basic metabolic panel, and oxygenation parameters. For each analyte, the 24 h change (Δ = T2 − T1) was analyzed alongside the T1 value to capture both baseline status and short-term physiological trajectory.

### 2.4. Outcomes

**Figure 1:**
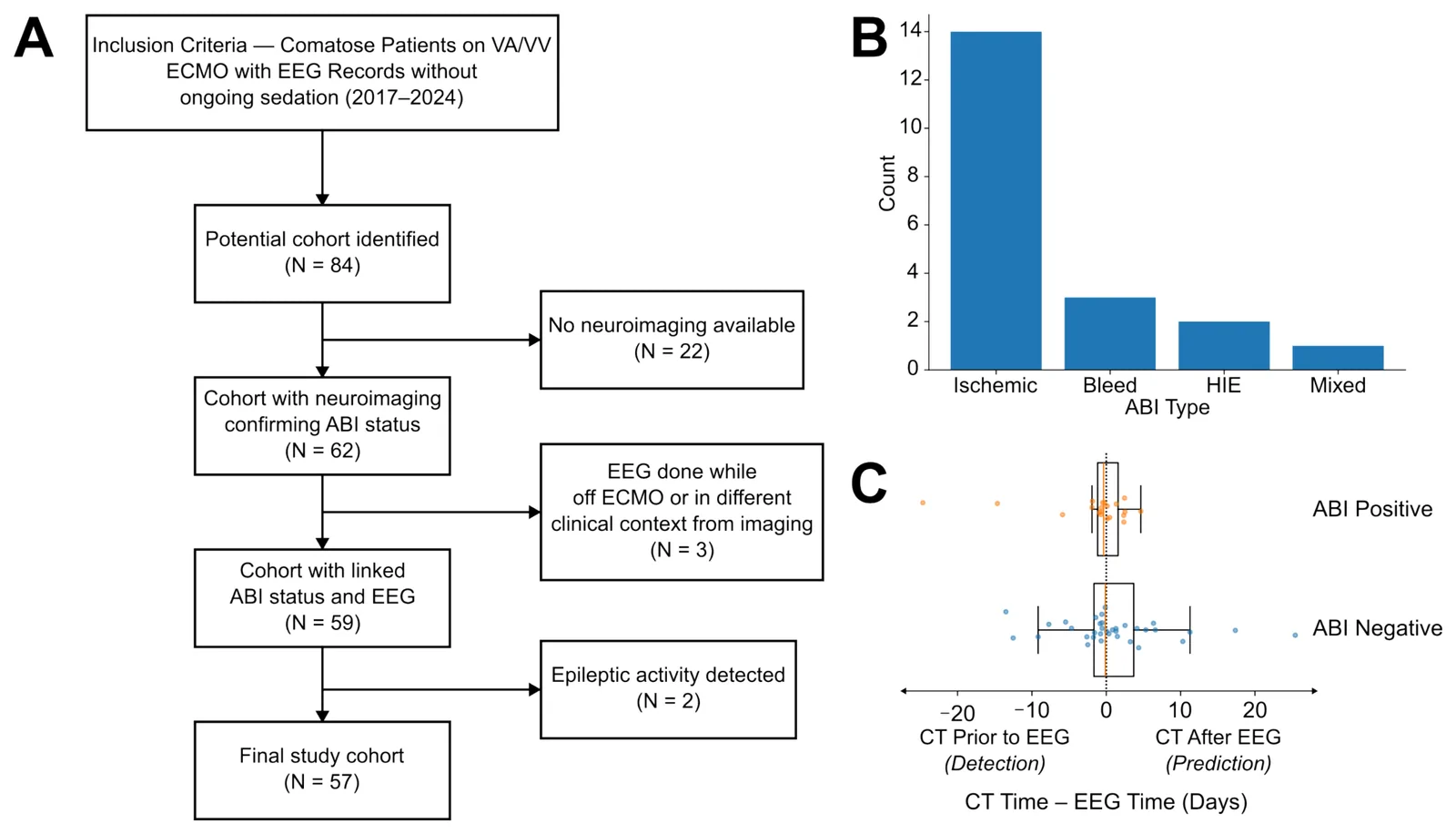
Study design and cohort characteristics. (**A**) Flow diagram illustrating patient selection, inclusion, and exclusion criteria. (**B**) Distribution of acute brain injury (ABI) subtypes identified by neuroimaging, including ischemic stroke, intracranial hemorrhage, hypoxic-ischemic injury, and mixed injury. (**C**) Distribution of the time interval between EEG acquisition and neuroimaging. Positive values indicate that neuroimaging was performed after EEG acquisition, and negative values that neuroimaging preceded EEG. Because the interval could fall on either side, the analysis reflects detection of concurrent ABI rather than prediction of subsequently developing injury. In panel (**C**), the vertical dotted line marks an interval of zero (neuroimaging and EEG on the same day); each point represents one patient; boxes indicate the interquartile range, orange lines the median, and whiskers the range of values within 1.5 times the interquartile range.

The primary outcome was ABI, defined as one or more neuroimaging findings during ECMO support: ischemic stroke, hypoxic-ischemic brain injury (HIBI), or intracranial hemorrhage (ICH; parenchymal, subarachnoid, subdural, or intraventricular). ABI was ascertained from neuroimaging (head CT and/or MRI) obtained closest in time to the EEG recording, in most patients within 72 h before or after the recording (Figure 1C), and adjudicated by neurocritical care physicians through structured chart review of radiology reports and clinical documentation. All referenced radiology interpretations had been formally read by attending neuroradiologists. When multiple subtypes were present, all were recorded, and ABI was analyzed as a binary outcome (present vs. absent). ABI during ECMO clusters early in the support course, frequently around the time of cannulation [7,9,27]. EEG obtained during protocolized sedation interruption therefore typically fell within the peri-injury window, and neuroimaging performed close in time to the EEG (Figure 1C) was treated as reflecting the patient’s brain-injury status at the time of recording rather than a future event. Thus, our model is more reflective of one for ABI detection rather than prediction. Patients were classified as ABI-negative when available neuroimaging showed no qualifying injury and no new focal neurological deficit was documented on the daily neurocritical care examination during the monitoring period. The secondary outcome was 30-day all-cause mortality, measured from the index EEG and ascertained from the electronic health record.

Coma was defined as a GCS ≤ 8 with no purposeful response to noxious stimulation. Lack of sedation was confirmed through a review of the patient’s Medication Administration Record at the time of EEG.

### 2.5. EEG Acquisition and qEEG Features

Continuous EEG was recorded using the international 10–20 electrode system at a sampling rate of 200 Hz. Signals were band-pass filtered with a fourth-order zero-phase Butterworth filter (0.5–40 Hz), after which segments contaminated by high electrode impedance or gross artifact were removed. The cleaned recordings were divided into artifact-free epochs for feature extraction.

From artifact-free epochs, 1169 quantitative EEG features spanning six domains (spectral power, interhemispheric asymmetry, nonlinear complexity, connectivity, suppression, and temporal trends) were computed and aggregated per patient as the median, interquartile range, and 95th and 99th percentiles. Feature definitions and the composition of each feature set are provided in Appendix A.

### 2.6. Classification Framework

Four EEG-only feature sets (an expert-curated 22-feature set, two data-driven Stability sets, and a theta-focused set), two clinical-only sets, and a combined set were compared; each outcome analysis draws on its matching Stability set, giving six sets per comparison. Full definitions appear in Appendix A. Seven base classifiers and three ensemble methods were trained with class-imbalance weighting. Full configurations are listed in Appendix B. An overview of the analysis pipeline is provided in Figure S1.

All models were evaluated by LOSO-CV, with imputation, scaling, and feature selection fitted only within each training fold to prevent data leakage. The two data-driven Stability sets were preselected once on the full cohort and are therefore interpreted as potentially optimistically biased relative to the fully nested sets. The primary metric was the area under the receiver operating characteristic (ROC) curve (AUC), with 95% confidence intervals from 1000 bootstrap resamples and the operating threshold set by Youden’s index. Differences in AUC between the best qEEG model and the clinical or combined models were tested with DeLong’s test for two correlated ROC curves evaluated on the patients common to both models, and paired differences in AUC were summarized with 2000-resample bootstrap 95% confidence intervals. Model calibration was assessed from the cross-validated out-of-fold predicted probabilities using the Brier score and the calibration intercept and slope obtained from the logistic recalibration $\mathrm{logit}\left(P\right)=a+b\;\mathrm{logit}\left(\hat{p}\right)$. Support vector machine probabilities were obtained by Platt scaling fitted within each training fold. To quantify the optimism and leakage introduced by defining the two data-driven Stability sets by doing feature selection on the entire dataset, we repeated their evaluation with the top-25 feature selection performed entirely within each training fold.

### 2.7. Statistical Analysis

Continuous variables are presented as the median [interquartile range, IQR] (age as mean ± standard deviation, SD) and were compared between patients with and without ABI using the Mann–Whitney U test, while categorical variables were compared using Fisher’s exact test. Multiple comparisons for laboratory variables were adjusted using the Benjamini–Hochberg false discovery rate procedure. All statistical tests were two-sided, and statistical significance was defined as p<0.05.

SHAP (SHapley Additive exPlanations) was applied to the best-performing single (non-ensemble) classifier, the random forest model, to decompose predictions into per-feature contributions [28]. Relative importance of EEG and clinical variables was additionally assessed with a random forest trained on the combined set, identifying the variables most associated with ABI detection and 30-day mortality. SHAP and Gini importances quantify each feature’s contribution to the output of the specific model in which it was computed. Given the small number of events and the high-dimensional feature space, they are interpreted throughout as model-specific feature contributions rather than evidence of clinically validated predictors.

Model training was performed in Python (version 3.12.2) using scikit-learn (v1.4.2), XGBoost (v3.0.1), and LightGBM (v4.6.0). Interpretability analysis was conducted with the SHAP (v0.51.0) package in Python [29,30,31].

## 3. Results

### 3.1. Patient Cohort

Among 57 comatose ECMO patients, 21 (37%) were diagnosed with ABI (Figure 1A, Table 1). The cohort had a mean age of 56 years and was 54% male; most were supported with venoarterial ECMO (41/57, 72%) for cardiac indications, and 83% were peripherally cannulated. At the time of EEG, all patients were off sedation, with a median GCS of 3 [3, 4] and RASS of −4 [−4, −3], consistent with the comatose inclusion criteria. Prior stroke or transient ischemic attack was uncommon. Among patients with ABI, injury was predominantly ischemic (70%), followed by hemorrhagic (15%), hypoxic-ischemic (10%), and mixed (5%) (Figure 1B; Table 2). The median interval from ECMO cannulation to EEG was 1.9 days in ABI-positive and 4.0 days in ABI-negative patients (Table 2). Overall, 30-day mortality was 51% and was higher among patients with ABI (70% vs. 40.5%). Most patients underwent confirmatory neuroimaging within 3 days of EEG (Figure 1C).

### 3.2. EEG Features Detect ABI Status

**Figure 2:**
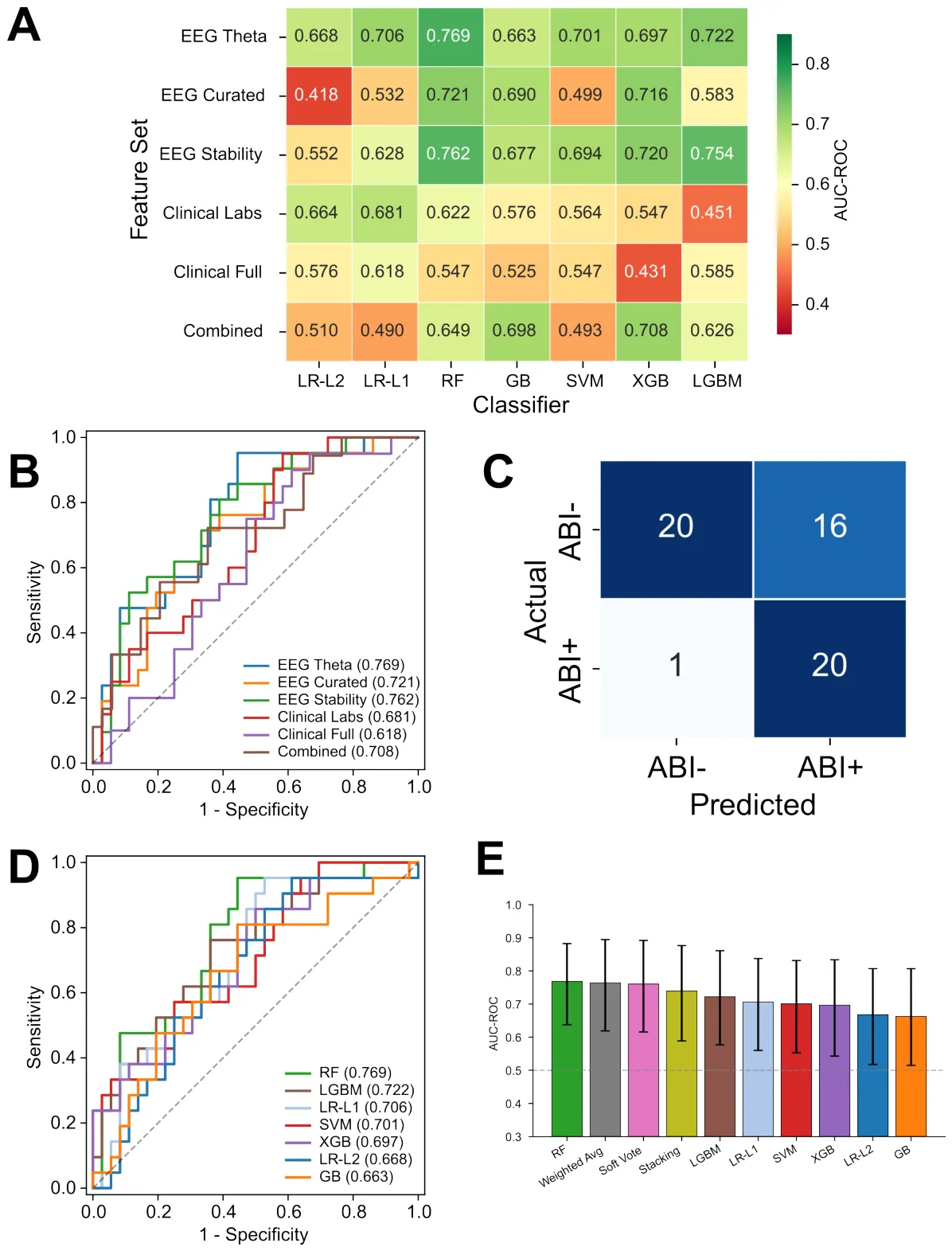
Quantitative EEG features improve ABI detection. (**A**) Heatmap of classifier performance across six feature sets evaluated using leave-one-subject-out cross-validation. EEG-derived feature sets consistently demonstrated higher AUC values than clinical variables alone. (**B**) ROC curves for the best-performing model from each feature set. (**C**) Confusion matrix for the highest-performing ABI detection model at the Youden-optimal threshold. (**D**) ROC curves comparing machine-learning classifiers trained on the EEG theta feature set. (**E**) Performance of individual and ensemble classifiers on the EEG theta feature set with bootstrap-derived 95% confidence intervals. The strongest discrimination was achieved using EEG features dominated by frontal theta activity, supporting the importance of EEG slowing as a marker of ABI during ECMO. Across panels, higher AUC indicates better discrimination. The overlapping confidence intervals in panel (**E**) indicate that differences between the best models were not statistically significant.

A total of 1169 quantitative EEG and 29 clinical features were extracted per patient and organized into six feature sets (Figure 2; Table 3). EEG-derived feature sets achieved higher point estimates than clinical variables alone for ABI detection, although their 95% confidence intervals overlapped. The highest-performing model was a theta-focused EEG set of 18 prespecified features (random forest), achieving an AUC of 0.769 (95% CI 0.638–0.883) versus 0.681 for clinical laboratory variables alone. In a paired comparison restricted to the patients evaluated by both models, this difference was not statistically significant (DeLong p=0.34; bootstrap ΔAUC +0.09, 95% CI −0.09 to +0.28), and no pairwise comparison among the feature sets reached significance. The theta model was reasonably calibrated (Brier score 0.19, calibration slope 0.89, intercept −0.22; Figure S2). Adding clinical variables to EEG features did not improve performance (AUC 0.708). Performance was consistent across EEG feature sets: a data-driven Stability set achieved an AUC of 0.762 and an expert-curated 22-feature set achieved 0.721. When the Stability set was instead reselected entirely within each cross-validation fold, its discrimination was not retained (AUC 0.48). The prespecified theta-focused and expert-curated sets are fixed feature lists and are not subject to this optimism, and they form the basis of our primary conclusion. These results suggest that qEEG may capture neurophysiological information relevant to ABI that is not fully available from routine clinical and laboratory variables.

### 3.3. EEG Features Most Important for ABI Detection

**Figure 3:**
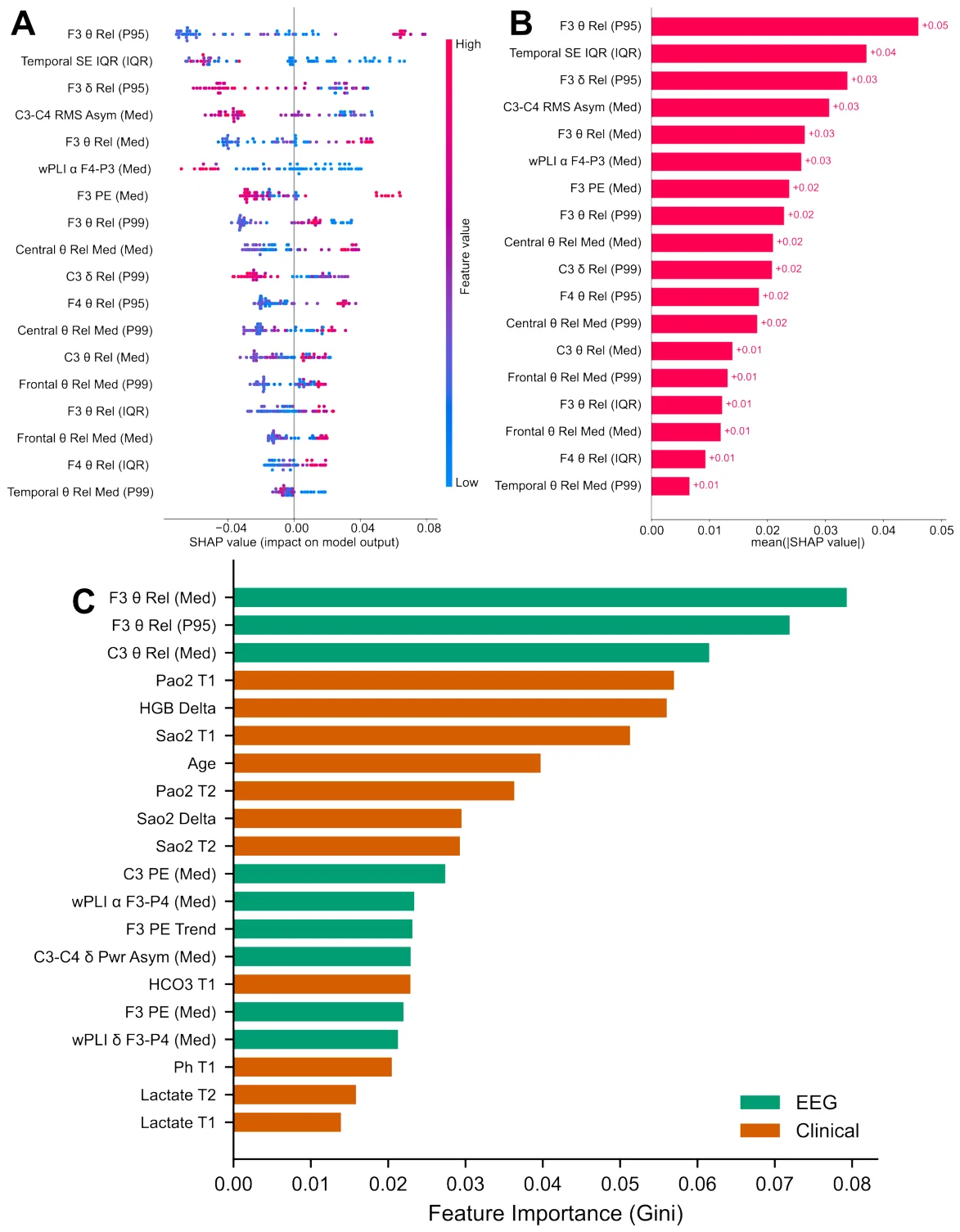
Feature importance in ABI detection. (**A**) SHAP beeswarm plot showing per-patient feature contributions for all 18 features of the EEG Theta set. Color indicates scaled feature value; horizontal position indicates SHAP contribution to prediction. (**B**) Mean |SHAP| values (bar plot) for the same 18 features of the random forest model, indicating overall feature importance for ABI detection. (**C**) Random forest feature importance (Gini) for the Combined Curated feature set, comparing EEG-derived (green) and clinical (orange) features. The top 20 features are shown.

SHAP analysis identified frontal theta activity as the dominant contributor to the model’s ABI predictions (Figure 3A,B). Relative theta power over the left frontal region (F3) was the most influential feature, followed by temporal Shannon entropy variability, frontal relative delta power, and interhemispheric asymmetry between C3 and C4. Overall, the highest-ranking features reflected two principal neurophysiological signatures of ABI, diffuse cortical slowing and hemispheric asymmetry.

In the combined EEG-clinical model, F3 relative theta power remained the highest-ranked feature (Figure 3C), followed by early oxygenation parameters (PaO_2_, arterial oxygen saturation, SaO_2_), 24 h hemoglobin change, and age, indicating that both cerebral electrophysiology and systemic physiological factors contributed to ABI detection. Dependence plots for the leading features are shown in Figure S3.

### 3.4. EEG and Clinical Features Predict 30-Day Mortality

**Figure 4:**
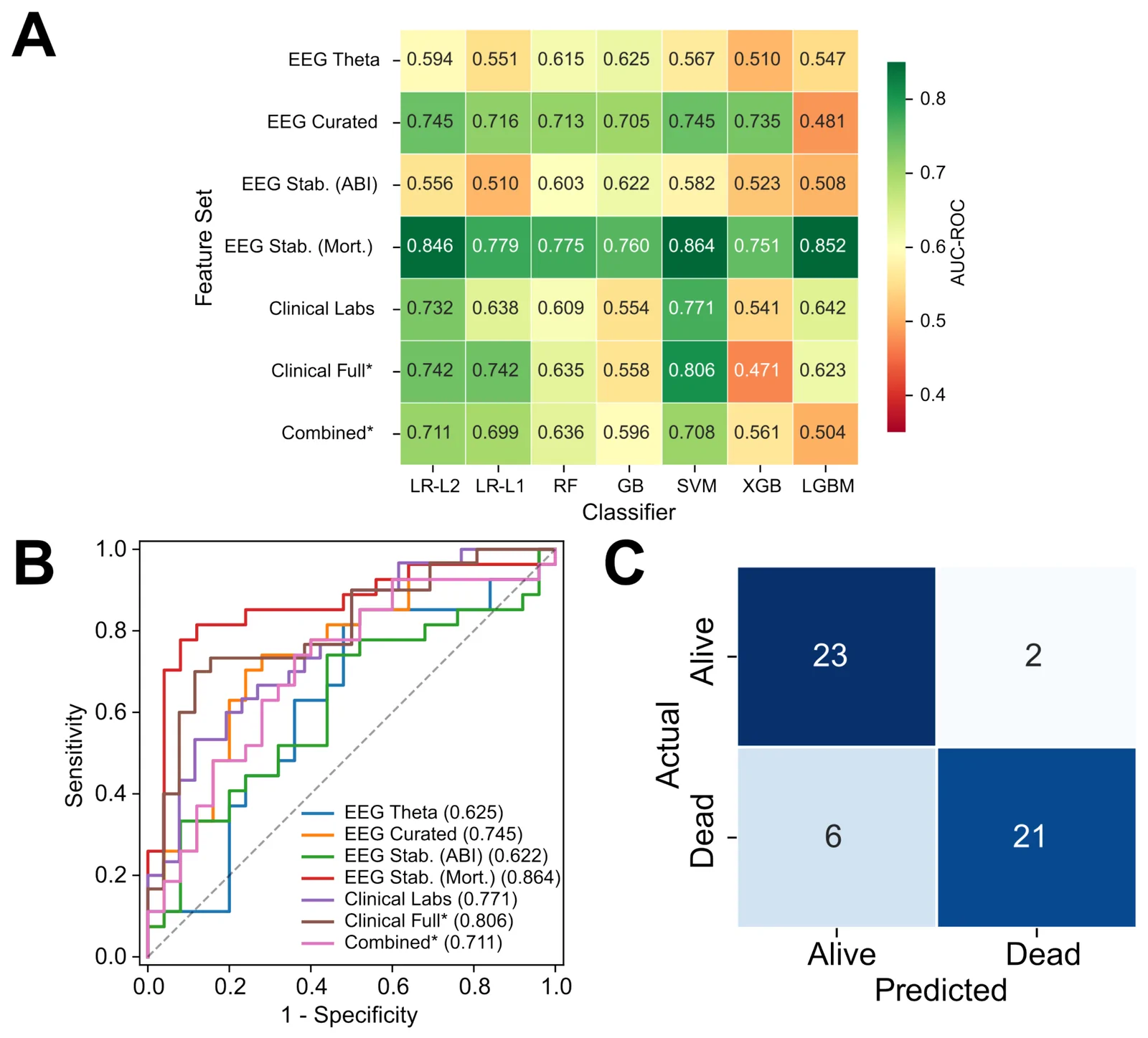
EEG features predict 30-day mortality. (**A**) AUC-ROC heatmap for seven feature sets (the six main feature sets plus the ABI-derived Stability set, shown for reference) across seven classifiers (LOSO-CV). (**B**) ROC curves for the best classifier on each of these feature sets. (**C**) Confusion matrix for the best model (SVM on EEG Stability (Mortality)) at the Youden-optimal threshold. Asterisks in panels (**A**,**B**) mark the feature sets that include non-laboratory clinical variables (age, sex, GCS, RASS, and ECMO configuration).

qEEG features also carried prognostic information for 30-day mortality (Figure 4). Among the EEG-only feature sets, discrimination ranged from an AUC of 0.745 for a prespecified expert-curated EEG feature set to 0.864 for a data-driven set. The best-performing model, a support vector machine (SVM) trained on the EEG Stability (Mortality) feature set, achieved an AUC of 0.864 and correctly classified 44 of 52 patients (sensitivity 77.8%, specificity 92.0% at the optimal threshold); mortality was evaluated in the 52 patients with an available 30-day outcome and complete clinical features. When feature selection was performed fully within each fold, discrimination fell to an AUC of 0.67. The prespecified expert-curated set (AUC 0.745) anchors our mortality interpretation, and the mortality model was well calibrated (Brier score 0.16, calibration slope 1.05, intercept −0.08; Figure S2). The most informative features included delta and theta relative power, alpha and beta activity, interhemispheric asymmetry, and entropy.

In paired comparisons, the mortality-optimized set did not significantly outperform the expert-curated set (DeLong p=0.07) or clinical laboratory variables (p=0.08). Combining EEG and clinical variables did not improve discrimination (AUC 0.711), suggesting that EEG-derived measures may reflect much of the prognostic information relevant to short-term survival.

## 4. Discussion

In this study, qEEG features achieved higher discrimination than routine clinical variables for the detection of ABI in comatose patients, suggesting that continuous neurophysiologic monitoring captures brain-specific information not fully reflected in conventional bedside assessments. Across multiple independently derived feature sets, frontal theta slowing, increased delta activity, reduced signal complexity, and interhemispheric asymmetry consistently emerged as the dominant electrophysiologic signatures of ABI. These same EEG-derived features also showed prognostic value for 30-day mortality, supporting qEEG as a candidate diagnostic and prognostic neuromonitoring tool during ECMO.

### 4.1. Frontal Theta Slowing and Interhemispheric Asymmetry Mark Acute Brain Injury

Diffuse slowing within the theta and delta frequency bands is a well-established marker of cortical dysfunction and has been associated with cerebral ischemia, hypoxic-ischemic injury, metabolic encephalopathy, and impaired consciousness [32,33,34,35]. Similarly, interhemispheric asymmetry reflects disruption of normal cortical organization and is commonly observed in focal structural lesions such as ischemic stroke and intracerebral hemorrhage, two of the most frequent neurological complications of ECMO [36]. The convergence of these features in our models suggests that qEEG captures both diffuse and focal manifestations of ABI.

Frontal theta activity, particularly over the left frontal region (F3), was the strongest contributor to ABI detection across all analyses. Although frontal theta slowing is generally considered a marker of diffuse cortical dysfunction [37], its left-sided predominance is biologically plausible in patients receiving peripheral VA-ECMO: the arterial mixing zone (i.e., Harlequin effect) between retrograde ECMO outflow and antegrade native output lies near the origin of the left common carotid, so the hemispheres are perfused with blood of differing oxygen content and pulsatility. Previous studies have demonstrated hemispheric differences in cerebral blood flow following ECMO cannulation [38], providing a potential hemodynamic explanation for the left-lateralized electrophysiologic signature, though this hypothesis will require simultaneous assessment with transcranial Doppler, cerebral oximetry, and neuroimaging.

Entropy-based features provided complementary information beyond conventional spectral analysis. Reduced EEG signal complexity has been linked to postanoxic coma and severe ABI, suggesting that nonlinear measures capture loss of normal cortical network dynamics not reflected by spectral power alone [39,40]. Similarly, interhemispheric asymmetry features, though secondary to frontal theta in SHAP ranking, were also meaningful contributors. Ischemic stroke, which accounts for a substantial proportion of ECMO-related ABI [27], produces focal or hemispheric disruption that manifests as asymmetric power distributions between homologous electrode pairs. The presence of C3–C4 root-mean-square (RMS) asymmetry among the top SHAP features is consistent with this lateralizing nature of ABI and aligns with prior work demonstrating the utility of asymmetry metrics in stroke detection from EEG [32,41]. These findings indicate that both diffuse network dysfunction and focal hemispheric injury contribute to the electrophysiologic phenotype of ABI during ECMO. Our model combined ischemic, hypoxic-ischemic, and hemorrhagic injuries into a single ABI outcome. However, these aggregate signatures should not be read as implying a common electrophysiologic mechanism across subtypes due to potential differential performance between these subtypes that cannot be fully evaluated in light of our limited cohort.

Interestingly, theta-band features were much less prominent for 30-day mortality; instead, beta-band features and interhemispheric asymmetry dominated the mortality-optimized feature set. The DINAMO study group, which investigated EEG features for prognostication in VA-ECMO patients, found that absence of alpha and beta bands only trended toward significance for predicting mortality [20]. However, a critical distinction is that our subjects were comatose without sedation, whereas the DINAMO study included patients on sedation, which is known to suppress higher frequencies [42]. This difference likely explains the greater discriminative value of beta-band features in our cohort.

### 4.2. qEEG Captures a Brain-Specific Signal Complementary to Clinical Prediction

Our findings extend previous work by suggesting that quantitative EEG provides complementary neurophysiologic information that is not fully captured by routine laboratory or hemodynamic variables. In pediatric ECMO, amplitude-integrated EEG asymmetry has been associated with cerebral injury, although prior studies evaluated only predefined indices without formal classification modeling [43]. In adult VA-ECMO, machine learning has been applied to high-temporal-resolution hemodynamic and clinical data to predict ABI (AUC 0.79) without incorporating neurophysiological data [24]. Our results indicate that qEEG identifies electrophysiologic signatures of brain injury while maintaining comparable discrimination.

An important methodological strength is the use of LOSO-CV with all preprocessing for the prespecified feature sets fitted exclusively on each training fold, providing a conservative and unbiased estimate of generalization performance. Many prior qEEG classification studies in critical care have used k-fold cross-validation without ensuring train-fold-only preprocessing, which can inflate reported performance through subtle data leakage [44,45]. Although the best-performing EEG model achieved only moderate discrimination (AUC 0.769), this performance likely represents a more realistic estimate of generalizability in a heterogeneous critically ill population.

The lack of improvement after adding clinical laboratory variables suggests that much of the predictive information contained within routine laboratory measurements is already reflected in cerebral electrophysiology. This observation is consistent with evidence that reduced-montage EEG provides physiological information not readily inferable from routine bedside variables in critically ill patients [46,47]. Nevertheless, the relatively small single-center cohort limits definitive conclusions regarding multimodal integration, and larger multicenter studies are needed to determine whether combining EEG with advanced physiologic or imaging biomarkers further improves prediction.

Integrating qEEG with other bedside neuromonitoring modalities is a natural next step. Circulating markers of brain injury, including glial fibrillary acidic protein, ubiquitin C-terminal hydrolase L1, neurofilament light chain, and tau, offer a molecular readout of injury complementary to the electrophysiologic signal [48,49,50], while transcranial Doppler and cerebral near-infrared spectroscopy add hemodynamic and oxygenation information [36], and ultra-low-field portable MRI increasingly allows structural confirmation at the bedside [17,18]. A multimodal framework combining qEEG with these measures may strengthen both detection and risk stratification and should be evaluated prospectively.

### 4.3. Clinical Implications and Future Directions

These findings have several practical implications for bedside neuromonitoring during ECMO. First, the identified feature hierarchy can guide the design of bedside EEG monitoring dashboards for ECMO patients. Displaying frontal theta trends, asymmetry, and entropy measures could give clinicians actionable quantitative information. As a sensitive screening tool, continuous qEEG may facilitate earlier identification of patients at high risk for ABI and prompt timely confirmatory neuroimaging or additional neuromonitoring.

Second, the prominence of frontal theta and asymmetry features suggests that reduced-montage EEG focused on these regions may retain much of the diagnostic information provided by conventional recordings [51]. Although prospective validation is required, this approach could enable scalable continuous neuromonitoring at centers without dedicated EEG expertise and inform channel selection for next-generation portable, wearable, and in-ear EEG systems designed for continuous neurological surveillance [52,53].

Third, seizures in comatose ECMO patients are frequently nonconvulsive and are undetectable on clinical examination under sedation or pharmacological paralysis, so continuous EEG is the principal means of identifying them [47,51]. Detecting these events, and titrating intravenous anesthetics to electrographic targets when status epilepticus becomes refractory [54], depends on expert review of the raw record and lies outside the scope of the summary qEEG features studied here.

### 4.4. Limitations

Several limitations should be acknowledged. First, the single-center retrospective design and modest sample size limit generalizability. The number of outcome events was small relative to the very large initial feature space, which raises a genuine risk of overfitting and model instability. The results are therefore best regarded as hypothesis-generating and require confirmation in larger cohorts. While LOSO-CV provides unbiased internal validation, external validation in an independent cohort is necessary before clinical translation. Because of the modest sample size, confidence intervals for all models were wide and overlapping, and formal comparisons between competing models did not reach statistical significance. The apparent performance advantage of qEEG requires confirmation in a larger, prospective, multicenter cohort with external validation before any clinical use.

Second, ABI adjudication from the medical record may be subject to ascertainment bias, as some brain injuries in ECMO patients go clinically unrecognized. Third, the qEEG features were aggregated over the entire monitoring period using summary statistics, providing a more binary discrimination of ABI rather than real-time detection. Future studies could leverage longitudinal EEG monitoring with time-windowed feature extraction to evaluate real-time detection performance. Fourth, because of the inclusion of EEG both before and after confirmatory neuroimaging, this study evaluates discriminative performance but does not establish whether qEEG changes temporally precede ABI diagnosis. Fifth, the ABI outcome combined ischemic, hypoxic-ischemic, and hemorrhagic injuries, which may carry different EEG signatures. The limited number of events precluded subtype-specific modeling, and the aggregate signatures should not be assumed to apply equally across these mechanistically distinct injuries. Sixth, a detailed neurological examination is often not feasible in comatose or pharmacologically paralyzed ECMO patients, so we could not formally test whether qEEG adds information beyond a structured bedside examination; a head-to-head comparison is an important direction for future work.

Finally, 30-day mortality reflects multiple non-neurological drivers (cardiogenic or respiratory failure, multi-organ dysfunction, withdrawal of care) and was not adjudicated by proximate cause; cause-of-death adjudication is needed, and the mortality models should not be interpreted as a specific measure of neurological prognosis.

## 5. Conclusions

In this retrospective cohort of comatose adult ECMO patients, quantitative EEG achieved higher discrimination than routine clinical variables for the detection of ABI and showed prognostic value for 30-day mortality, although these differences did not reach statistical significance given the available sample size. Frontal theta slowing, reduced signal complexity, and interhemispheric asymmetry emerged as reproducible electrophysiologic signatures of brain injury across the prespecified feature sets, supporting their biological relevance and robustness. These findings suggest that qEEG captures brain-specific information beyond conventional clinical assessment and motivate prospective, multicenter studies with external validation, reduced-montage continuous monitoring, and integration into automated bedside neuromonitoring systems for patients receiving ECMO.

## Figures and Tables

**Table 1:** Baseline demographic and ECMO characteristics of the study cohort.

Characteristic	ABI (*n* = 21)	No ABI (*n* = 36)	*p*-Value
*Demographics*			
Age, mean ± SD	59.5 ± 12.0	54.2 ± 15.1	0.161
*Sex*			
Male	60.0%	51.4%	0.729
Female	40.0%	48.6%	
*Race*			
Asian	0%	2.7%	0.144
Black	35.0%	43.2%	
Hispanic	0%	8.1%	
White	40.0%	35.1%	
Middle Eastern	5.0%	0%	
Other	5.0%	0%	
Unknown	15.0%	8.1%	
*ECMO characteristics*			
Duration, days [IQR]	8.5 [5.3, 14.4]	10.7 [5.3, 22.5]	0.617
*Indication*			
Pulmonary support	10.0%	35.3%	0.079
Cardiac support	65.0%	47.1%	
ECPR	25.0%	17.6%	
*Configuration*			
VA	80.0%	67.6%	0.163
VV	20.0%	29.7%	
VA-V	0%	2.7%	
*Cannulation site*			
Central	20.0%	14.7%	0.767
Peripheral	80.0%	85.3%	

ABI, acute brain injury; ECMO, extracorporeal membrane oxygenation; ECPR, extracorporeal cardiopulmonary resuscitation; VA, venoarterial; VV, venovenous; VA-V, venoarterial-venous; SD, standard deviation; IQR, interquartile range. Continuous variables are reported as mean ± SD or median [IQR]; categorical variables as percentages. *p*-values are from the Mann–Whitney U test (continuous) or Fisher’s exact test (categorical). Descriptive statistics in Table 1 and Table 2 were computed with the imaging-subtype-documented classification (20 patients with ABI, 37 without); one additional patient was classified as ABI-positive for the modeling analyses (21 with ABI, 36 without; Table 3 and Figure 2, Figure 3 and Figure 4), in which feature sets containing clinical variables were evaluated in the patients with complete clinical data. Percentages are computed on the patients with the variable available, so per-variable denominators may be smaller. Race percentages in the No ABI column sum to 97.2% because race was not documented for one patient. Italic row headings denote variable categories.

**Table 2:** Neurological status, EEG characteristics, and clinical features at the time of EEG.

Variable	ABI+ (*n* = 21)	ABI− (*n* = 36)	*p*-Value
*Neurological status*			
GCS	3 [3, 4]	3 [3, 3]	0.090
RASS	−4 [−4, −2]	−4 [−4, −3]	0.811
*ABI subtype (among ABI+)*			
Ischemic	70.0%	N/A	
Hemorrhagic	15.0%	N/A	
Mixed	5.0%	N/A	
Hypoxic-ischemic brain injury	10.0%	N/A	
*EEG characteristics*			
EEG duration, h	27.8 [24.2, 48.2]	36.5 [25.3, 47.8]	0.519
Time from ECMO cannulation to EEG, days	1.9 [0.7, 3.7]	4.0 [1.2, 6.7]	0.104
Time from EEG to CT, days	−0.15 [−1.65, 3.68]	−0.36 [−1.15, 1.60]	0.155
*Outcome*			
30-day mortality	70.0%	40.5%	0.065
*Laboratory values at EEG*			
Lactate, mmol/L	2.0 [1.34, 3.00]	1.8 [1.1, 2.8]	0.760
PaCO_2_, mmHg	35 [34, 41]	38 [33, 45]	0.522
PaO_2_, mmHg	154.5 [84, 258]	82 [67, 137]	**0.013**
Hemoglobin, g/dL	8.2 [7.8, 9.6]	8.5 [7.8, 9.4]	0.481
Platelets, ×10^3^/μL	62 [49, 103]	68 [52, 84]	0.355

ABI, acute brain injury; GCS, Glasgow Coma Scale; RASS, Richmond Agitation–Sedation Scale; EEG, electroencephalography; CT, computed tomography; PaCO_2_, partial pressure of carbon dioxide; PaO_2_, partial pressure of oxygen; N/A, not applicable. Continuous variables are reported as median [IQR]. *p*-values are from the Mann–Whitney U test (continuous) or Fisher’s exact test (categorical); bold denotes p<0.05. Laboratory *p*-values are unadjusted; after Benjamini–Hochberg adjustment across the laboratory variables, no laboratory difference remained statistically significant. ABI subtype percentages are among ABI-positive patients with a documented imaging subtype. As in Table 1, descriptive statistics were computed with the imaging-subtype-documented classification (20 with ABI, 37 without). Per-variable denominators may be smaller where data were unavailable. Italic row headings denote variable categories.

**Table 3:** ABI classification performance of the best classifier for each main feature set (LOSO-CV).

Feature Set	*N*	Best Classifier	AUC (95% CI)	Sensitivity	Specificity
EEG Stability	25	Random Forest	0.762 (0.619–0.890)	0.810	0.611
**EEG Theta**	**18**	**Random Forest**	**0.769 (0.638–0.883)**	**0.952**	**0.556**
EEG Curated	22	Random Forest	0.721 (0.580–0.848)	0.714	0.667
Clinical Labs	16	LogReg L1	0.681 (0.533–0.815)	0.950	0.417
Clinical Full	29	LogReg L1	0.618 (0.467–0.762)	0.900	0.389
Combined Curated	51	XGBoost	0.708 (0.539–0.848)	0.722	0.647

The best-performing model overall (EEG Theta) is shown in bold. AUC, area under the receiver operating characteristic curve; CI, confidence interval; LOSO-CV, leave-one-subject-out cross-validation; LogReg L1, L1-regularized logistic regression. Confidence intervals were derived from 1000 bootstrap resamples; the operating threshold was set by Youden’s index.

## Data Availability

The data presented in this study are available upon reasonable request from the corresponding author. The data are not publicly available due to privacy and ethical restrictions concerning sensitive patient information.

## Supplementary Materials

The following supporting information can be downloaded at https://www.mdpi.com/article/10.3390/jcm15176761/s1, Supplementary Methods: full inclusion and exclusion criteria; Figure S1: Schematic overview of the analysis pipeline; Figure S2: Calibration curves for the primary ABI and mortality models (Brier score, calibration slope, and intercept); and Figure S3: SHAP dependence plots for the leading features.

## Abbreviations

The following abbreviations are used in this manuscript:

ABI	Acute brain injury
AUC	Area under the receiver operating characteristic curve
cEEG	Continuous electroencephalography
CI	Confidence interval
CT	Computed tomography
ECMO	Extracorporeal membrane oxygenation
ECPR	Extracorporeal cardiopulmonary resuscitation
EEG	Electroencephalography
GCS	Glasgow Coma Scale
HIBI	Hypoxic-ischemic brain injury
ICH	Intracranial hemorrhage
IQR	Interquartile range
LOSO-CV	Leave-one-subject-out cross-validation
MRI	Magnetic resonance imaging
qEEG	Quantitative electroencephalography
RASS	Richmond Agitation–Sedation Scale
RMS	Root mean square
ROC	Receiver operating characteristic
SHAP	SHapley Additive exPlanations
SVM	Support vector machine
VA	Venoarterial
VA-V	Venoarterial-venous
VV	Venovenous

## Appendix A. Feature Set Definitions

Quantitative EEG and clinical features were organized into the feature sets summarized in Table A1. The composition of each EEG feature set is detailed in Table A2, Table A3, Table A4 and Table A5, and the clinical feature sets are detailed in Table A6.

**Table A1:** Summary of feature sets.

Feature Set	*N*	Category	Description
EEG Curated	22	EEG	Expert-selected features across six qEEG domains (see Table A2)
EEG Stability (ABI)	25	EEG	Top 25 features by random forest importance, selected once on the full cohort, trained on the presence of ABI (see Table A3)
EEG Stability (Mortality)	25	EEG	Top 25 features by random forest importance, selected once on the full cohort, trained on 30-day mortality (see Table A4)
EEG Theta	18	EEG	Theta-centric minimal set: relative theta and delta power, entropy, asymmetry, and connectivity (see Table A5)
Clinical Labs	16	Clinical	Lab values at time of EEG (T1) and 24 h after (T2): lactate, PaCO_2_, PaO_2_, SaO_2_, pH, HCO_3_, hemoglobin, platelets (see Table A6)
Clinical Full	29	Clinical	Labs T1/T2 + (T2 − T1) deltas + age, sex, GCS, RASS, ECMO type (VA/VV) (see Table A6)
Combined Curated	51	Combined	EEG Curated (22) + Clinical Full (29)

**Table A2:** EEG Curated feature set.

Domain	*N*	Features
Asymmetry	5	C3–C4 alpha power (median, IQR), C3–C4 delta power (median), F3–F4 alpha power (IQR), O1–O2 alpha power (median) [55]
Suppression	5	Global suppression max (median, IQR, P95), global suppression mean (IQR), % epochs with >50% channel suppression [56]
Theta/entropy	5	F3 relative theta power (median, P95), C3 relative theta power (median) [57], F3 permutation entropy (median), C3 permutation entropy (median) [58]
Connectivity	2	Weighted phase lag index (wPLI) [59]: alpha F3–P4 (median), delta F3–P4 (median)
Spectral power	3	C3 absolute delta power (median), F3 absolute alpha power (median), frontal alpha-delta ratio (median) [60]
Temporal trends	2	Linear trend of F3 permutation entropy, linear trend of global suppression mean [56]

Per-patient summary statistics of the epoch-wise values are given in parentheses: median; IQR, interquartile range; P95 and P99, 95th and 99th percentiles (Table A2, Table A3, Table A4 and Table A5). RMS, root mean square; wPLI, weighted phase lag index.

**Table A3:** EEG Stability (ABI) feature set.

Domain	*N*	Details
Relative theta power	14	F3 (median, P95, P99), C3 (median, P95), C4 (P95), P3 (P99), frontal regional (median-median, median-P95, median-P99), central regional (median-P95, median-P99), occipital regional (median-P95, IQR-P99)
Relative delta power	4	F3 (P95), C3 (P99), central regional (median-P99), temporal regional (median-P99)
Asymmetry	3	C3–C4 RMS (median), C3–C4 beta power (median), O1–O2 RMS (IQR)
Shannon entropy	2	Occipital regional (IQR-P99), temporal regional (IQR-IQR)
Alpha/delta ratio	1	Occipital regional (median-IQR)
RMS amplitude	1	Occipital regional IQR (P99)

**Table A4:** EEG Stability (Mortality) feature set.

Domain	*N*	Details
Absolute beta power	13	F4 (median), C3 (median), C4 (median), P3 (median), frontal regional (median-median, IQR-median, IQR-P95), central regional (median-median), temporal regional (median-median, IQR-median, IQR-P95, IQR-IQR), occipital regional (IQR-IQR)
Asymmetry (RMS, delta)	5	T3–T4 RMS (median), T3–T4 delta power (median), P3–P4 RMS (median), P3–P4 delta power (median), C3–C4 delta power (median)
Relative delta power	2	Temporal regional (IQR-P95, IQR-P99)
Entropy/trend	2	Frontal Shannon entropy (IQR-P95), parietal permutation-entropy trend (IQR)
Total power	1	Temporal regional (IQR-IQR)
Absolute alpha power	1	C3 (IQR)
Relative theta power	1	Occipital regional (IQR-median)

**Table A5:** EEG Theta feature set.

Domain	*N*	Features
Relative theta power	12	F3 (IQR, median, P95, P99), F4 (P95, IQR), C3 (median), frontal regional (median, P99), central regional (median, P99), temporal regional (P99)
Relative delta power	2	C3 (P99), F3 (P95)
Entropy	2	Temporal Shannon entropy (IQR), F3 permutation entropy (median)
Asymmetry	1	C3–C4 RMS asymmetry (median)
Connectivity	1	wPLI alpha F4–P3 (median)

**Table A6:** Clinical feature sets (Clinical Labs and Clinical Full).

Category	*N*	Variables
Laboratory values (T1, T2)	16	Lactate, PaCO_2_, PaO_2_, SaO_2_, pH, HCO_3_, hemoglobin, platelets, each measured at T1 and T2
Laboratory change (Δ = T2 − T1)	8	24-h change for each of the eight laboratory values
Demographic and clinical	5	Age, sex, GCS at EEG, RASS, ECMO type (VA/VV)

The Clinical Labs feature set comprises the 16 laboratory values (the eight analytes above, each at T1 and T2). The Clinical Full feature set comprises all 29 variables (16 laboratory values + 8 laboratory changes + 5 demographic and clinical variables). T1, closest to EEG initiation; T2, approximately 24 h later. SaO_2_, arterial oxygen saturation; HCO_3_, bicarbonate.

## Appendix B. Model Configurations and Hyperparameters

The configurations and hyperparameters for all base classifiers, ensemble methods, and preprocessing steps are summarized in Table A7.

**Table A7:** Model configurations and hyperparameters.

Component	Configuration
Logistic regression	L1 and L2 regularization; balanced class weights
Random forest	500 trees; balanced class weights
Gradient boosting	50 trees
Support vector machine	RBF kernel; balanced class weights
XGBoost	Gradient-boosted trees; class-imbalance weighting
LightGBM	Gradient-boosted trees; class-imbalance weighting
Ensembles	Soft voting, AUC-weighted averaging, and stacking (logistic-regression meta-learner on out-of-fold predictions), built on the top 3 classifiers per feature set
Preprocessing	Median imputation; standard scaling
Feature selection	Variance thresholding; correlation filter |r|>0.95; univariate ANOVA F-test, top 25 features
Cross-validation	Leave-one-subject-out
Operating threshold	Youden’s index
Confidence intervals	Bootstrap resampling, 1000 iterations
