# Supplementary material for: Critical Care Monitoring in Comatose Patients Receiving ECMO: A Cohort Study of Machine Learning on Quantitative EEG for Diagnosing Acute Brain Injury and Prognosticating Mortality
Source: J Clin Med. 2026 Aug 31;15(17):6761. doi: 10.3390/jcm15176761 (PMC13566830; doi:10.3390/jcm15176761)
Supplement: Supplementary file 1 [file jcm-15-06761-s001.zip › jcm-4466001-supplementary.pdf]

Supplementary Methods

Patient Eligibility Criteria. The following inclusion and exclusion criteria defined the analysis cohort. Inclusion criteria were: (i) adult VA, VV, or hybrid VA-V ECMO support during the study period; (ii) at least one continuous EEG recording of ≥12 uninterrupted hours (sessions were not concatenated to meet this threshold); and (iii) brain neuroimaging (head CT and/or MRI) permitting ABI ascertainment. For patients with multiple ECMO runs or qualifying recordings during the same admission, only the index recording (the EEG session most proximal to the qualifying neuroimaging) was analyzed, to avoid intra-patient correlation. Exclusion criteria were: (i) documented epileptiform activity during monitoring, including status epilepticus (convulsive or non-convulsive), electrographic seizures, or repetitive periodic discharges meeting ictal criteria (adjudicated by the clinical neurology team), because such patterns confound qEEG stability metrics; and (ii) absence of qualifying neuroimaging.

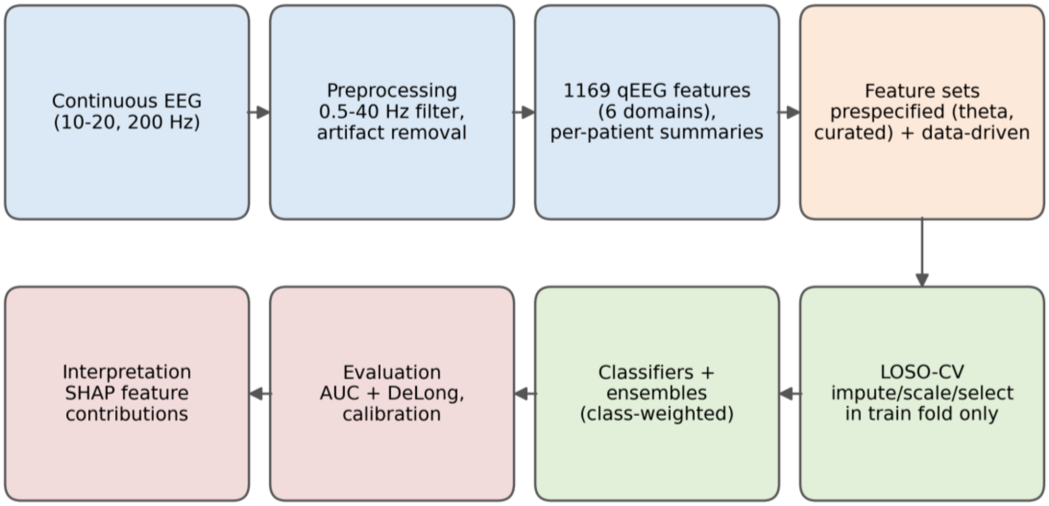

Figure S1. Schematic overview of the analysis pipeline: continuous EEG acquisition, preprocessing and artifact removal, extraction of 1169 quantitative EEG features, definition of prespecified and data-driven feature sets, leave-one-subject-out cross-validation with all preprocessing fitted within the training fold, model training with class-imbalance weighting, and evaluation by discrimination (AUC, DeLong), calibration, and SHAP.

Supplementary Results

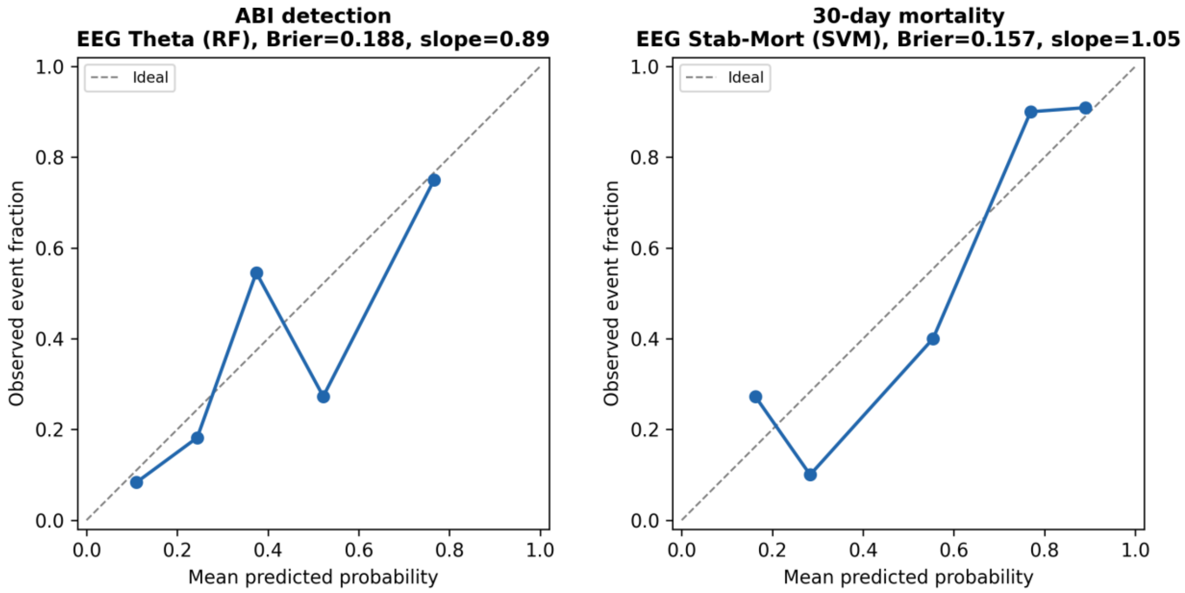

Figure S2. Calibration (reliability) curves for the primary ABI model (EEG Theta, Random Forest) and the primary 30-day mortality model (EEG Stability, SVM with within-fold Platt scaling), from leave-one-subject-out out-of-fold probabilities. Brier scores were 0.19 (ABI) and 0.16 (mortality); calibration slopes were 0.89 and 1.05 (ideal 1.0) with intercepts −0.22 and −0.08. The dashed line denotes perfect calibration.

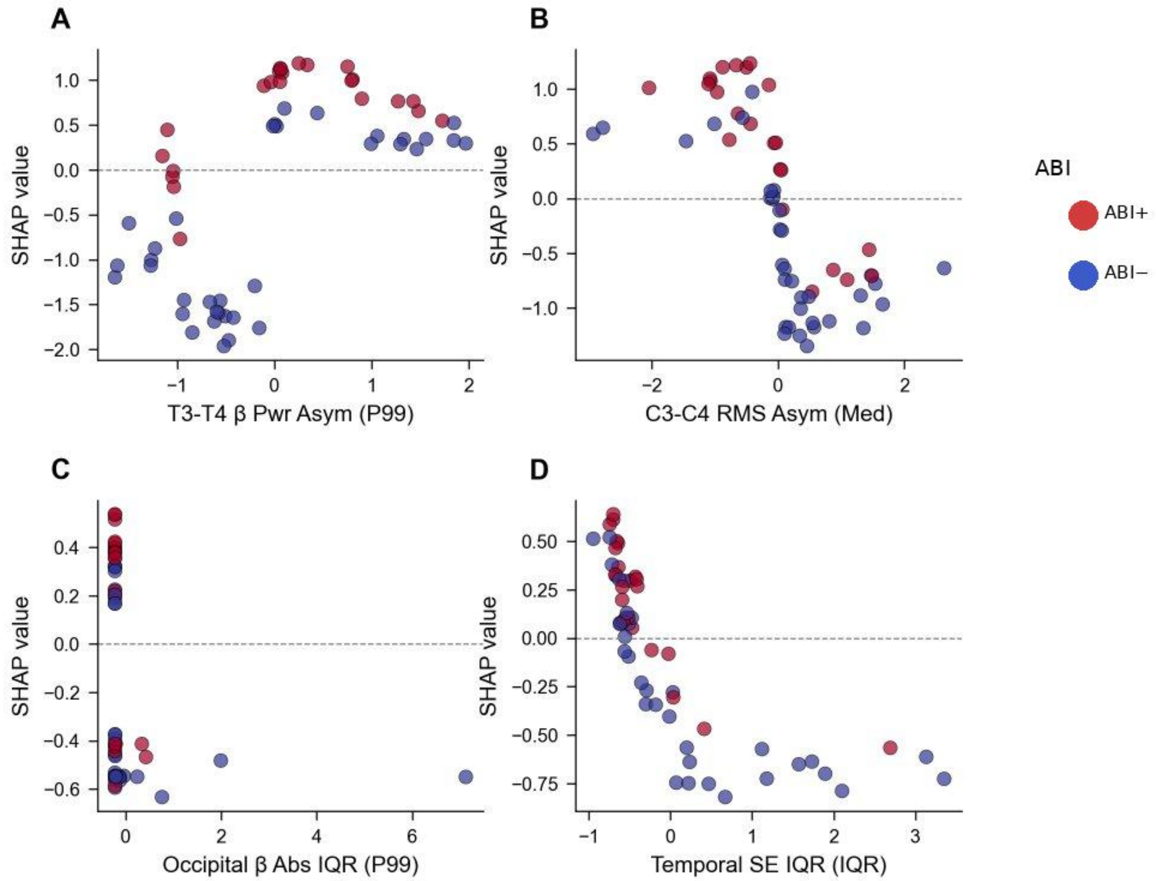

Figure S3. SHAP dependence plots for the top four features (A–D), illustrating the nonlinear and non-monotonic relationships between feature values and model predictions. Color indicates ABI status.
